# Up-Regulation of *miR-21* Is Associated with Cervicitis and Human Papillomavirus Infection in Cervical Tissues

**DOI:** 10.1371/journal.pone.0127109

**Published:** 2015-05-26

**Authors:** Sureewan Bumrungthai, Tipaya Ekalaksananan, Mark Francis Evans, Peechanika Chopjitt, Thumwadee Tangsiriwatthana, Natcha Patarapadungkit, Pilaiwan Kleebkaow, Sanguanchoke Luanratanakorn, Bunkerd Kongyingyoes, Suchin Worawichawong, Chamsai Pientong

**Affiliations:** 1 Department of Microbiology, Faculty of Medicine, Khon Kaen University, Khon Kaen, Thailand; 2 Department of Pathology and Laboratory Medicine, University of Vermont College of Medicine, Burlington, Vermont, United States of America; 3 Department of Obstetrics and Gynecology, Khon Kaen Hospital, Khon Kaen, Thailand; 4 Department of Pathology, Faculty of Medicine, Khon Kaen University, Khon Kaen, Thailand; 5 Department of Obstetrics and Gynecology, Faculty of Medicine, Khon Kaen University, Khon Kaen, Thailand; 6 Department of Pharmacology, Faculty of Medicine, Khon Kaen University, Khon Kaen, Thailand; 7 Department of Pathology, Faculty of Medicine, Ramathibodi Hospital, Mahidol University, Bangkok, Thailand; 8 HPV & EBV and carcinogenesis Research Group, Khon Kaen University, Khon Kaen, Thailand; The University of Hong Kong, Queen Mary Hospital, HONG KONG

## Abstract

*MicroRNA-21* (*miR-21*) is recognized as an oncomir and shows up-regulation in many types of human malignancy. The aim of this study was to investigate the association of *miR-21* expression associated with HPV infection in normal and abnormal cervical tissues. Cervical tissue samples with different cytological or histopathological grades were investigated for HPV by PCR and for *miR-21* and programmed cell death, protein 4 (*PDCD4*) expression using quantitative real-time PCR (qRT-PCR). Laser capture microdissection (LCM) of stromal and epithelial tissues and *in situ* hybridization (ISH) using locked nucleic acid (LNA) probes were performed on a subset of fixed specimens. Cell line experiments were conducted on fibroblasts stimulated in culture media from HeLa cells, which were then assessed for *miR-21*, *PDCD4*, *IL-6* and α-*SMA* expression by qRT-PCR. Twenty normal cervical cell, 12 cervicitis, 14 cervical intraepithelial neoplastic I (CIN I), 22 CIN II-III and 43 cervical squamous cell carcinoma (SCC) specimens were investigated. *miR-21* levels were significantly lower in normal than in abnormal tissues. The expression of *miR-21* in HPV negative normal cytology was significantly lower than in HPV positive samples in abnormal tissue and SCC. The *miR-21* expression was significantly higher in HPV negative cervicitis than HPV negative normal cells. LCM and ISH data showed that *miR-21* is primarily expressed in the tumor-associated stromal cell microenvironment. Fibroblasts treated with HeLa cell culture media showed up-regulated expression of *miR-21*, which correlated with increased expression of α-*SMA* and *IL-6* and with down-regulation of *PDCD4*. These results demonstrate that *miR-21* is associated with HPV infection and involved in cervical lesions as well as cervicitis and its up-regulation in tumor-stroma might be involved in the inflammation process and cervical cancer progression.

## Introduction

Cervical cancer is the second most common malignancy in women worldwide. High-risk human papillomavirus (HR-HPV) infection is recognized as the most important risk factor. Chronic over-expression of the HPV *E6* and *E7* oncogenes promote tumor progression by inducing genetic and epigenetic instability [1–3].

Epigenetic instability is impacted by microRNAs (*miRNA* or *miR-*). *miR-* dysregulation is associated with a wide variety of human malignancies. Through 3’-UTR binding of the target-mRNA, *miRs* repress gene translation [4]. Several *miRs* are reported to be involved in cervical cancer such as *miRs-21*, *-23b*, *-34a*, *-143*, *-146a*, *-218* and *miR-182*; these *miRs* play crucial roles in cervical cell proliferation, differentiation and apoptosis [5–13].

*miR-21* is a regulator of gene expression at the post-transcriptional level, and is increased in many types of human malignancies including colon [14], pancreatic [15], breast, prostate [16], oral [17], ovarian [18] and cervical [7–9, 19] cancers. *miR-21* is known to promote cell proliferation [7] and initiate inflammation-associated carcinogenesis via nuclear factor kappa-light-chain-enhancer of activated B cells (NF-kB) and interleukin-6 (IL-6) signaling pathways in colon and cervical cancer cells [20, 21]. The genes of programmed cell death, protein 4 (*PDCD4*) [7, 19] and phosphatase and tensin homolog (*PTEN*), are known as the targets of *miR-21* [16]. The regulatory region of *miR-21* gene consists of several binding sites for transcription factors such as activator protein 1 (AP-1), and signal transducer and activator of transcription 3 (STAT3) [20].

*miR-21* expression is up-regulated in cervical cancer cell lines, especially in HPV16-positive CaSki cells and HPV18-positive HeLa cells [8, 9]. The up-regulation of *miR-21* promotes cell proliferation and down-regulation of PDCD4 [7]. Moreover, *miR-21* qRT-PCR expression levels correlate with the histological staging of cervical cancer, and so might be used as a screening marker [19]. High *miR-21* levels have been reported in the tumor-stromal compartment of colorectal cancers: *miR-21* expression is associated with the progression and short disease-free survival in patients with stage II colon cancer [14, 22]. The importance of tumor-stromal fibroblast expression patterns in tumor progression is well recognized; for example, stromal fibroblasts promote tumor growth and angiogenesis in pancreatic cancer [23] and invasive human breast carcinomas [24]. The aberrant expression of *miR-21* in tumor-stromal cells may therefore be associated with carcinogenesis [25].

Fibroblasts are the major component cell type in connective tissues. They are involved in the production of growth factors, chemokines and the extracellular matrix and differentiate to perform altered functions in response to cytokines and other protein signals. IL-6 is a pleiotropic cytokine that is involved in several stages of tumor development and can mediate epithelial-stromal interactions [26]. IL-6 up-regulation has been implicated as a significant element in cervical cancer pathogenesis by a number of studies [27–33]. IL-6 has been frequently detected in the stromal region of cervical cancer tissues containing a large number of fibroblasts [21, 34]. Additionally, IL-6 positive cells in the stromal cells show positive staining for the fibroblast marker α-smooth muscle actin (α-*SMA*) [26]. The α-*SMA* together with IL-6 and transforming growth factor-β (TGF-β) are involved in (myofibroblast differentiation by up-regulation of *miR-21* by interaction with PDCD4 in the tumor-stroma [25].

To date, there have been few studies of *miR-21* in cervical cancer [7–9, 19]; the aim of the present study was the investigation of *miR-21* in cervical carcinogenesis in relation to HPV infection and apoptotic functioning. *miR-21* expression in normal cervical cells and fresh cervical tissues with different histopathological grades was evaluated using quantitative real-time PCR (qRT-PCR). The localization of *miR-21* expression was investigated in formalin-fixed, paraffin-embedded (FFPE) sections by qRT-PCR comparison of cervical epithelium and stromal region RNA extracts isolated by laser capture microdissection (LCM) by qRT-PCR and by *in situ* hybridization (ISH). HPV status in fresh biopsies was demonstrated by PCR. Additionally, the induction of *miR-21* in fibroblasts was investigated by treatment of the amniotic fibroblasts with culture medium from HeLa cells (HeLa CM) and TGF-β1 following: α*-SMA*, *miR-21*, *PDCD4* and *IL-6* levels were measured by qRT-PCR.

## Materials and Methods

### Patients and tissues

Cervical samples including normal cervical cells and cervical tissues were collected from women who underwent routine cervical cancer investigation by Papanicolaou (Pap) smear testing in combination with colposcopy at Srinagarind Hospital and Khon Kaen Hospital, Khon Kaen, Thailand and provided their written informed consent to participate in previous projects approved by the Human Research Ethics Committee of Khon Kaen University and Khon Kaen Hospital. This work used leftover specimens from the previous projects and was approved by the Human Research Ethics Committee of Khon Kaen University (HE541168). The requirement for written informed consent was waived by the committee for reasons including the use of archival specimens collected prior to study initiation. The specimens were grouped according to the histological diagnosis reviewed by the pathologist. Samples used in this study consisted of normal cytology diagnosed by Pap smear (20 samples), fresh cervical tissues obtained at colposcopy (91 samples) and FFPE cervical tissues (70 samples). The fresh cervical tissues (91 samples) were classified histopathologically into cervicitis (12 cases), cervical intraepithelial neoplasia-I (CIN I) (14 cases), CIN II-III (22 cases) and cervical squamous cell carcinoma (SCC) (43 cases). The FFPE cervical tissues consisted of cervititis (n = 26), CIN I (n = 11), CIN II-III (n = 21) and SCC (n = 12).

### RNA and DNA extraction

RNA and DNA were extracted from normal cervical cells, cervical tissues and FFPE samples (cervical lesions and stromal region) using the Trizol reagent kit (Ambion, Life Technology, Carlsbad, CA, USA) according to manufacturer’s instructions. For RNA extraction from FFPE, tissues were deparaffinized with xylene and rehydrated with serial ethanol and air-dried before experiments. Cells and tissues samples were mixed with Trizol reagent, homogenized and incubated for 5 min at 15–30°C. Chloroform (0.2 ml) was added to the homogenized solution (per 1 ml) and then the sample tubes were shaken for 2–3 min at 15–30°C and centrifuged at 12,000x g for 15 min at 2–8°C. The aqueous phase was transferred to a fresh tube and the organic phase was collected for DNA isolation. For precipitation of the RNA, isopropyl alcohol (0.5 ml) was added to 1 ml Trizol aqueous phase, mixed, incubated for 10 min at 15–30°C and centrifuged at 12,000x g for 10 min at 2–8°C. The supernatant was removed and with 1 ml of 75% ethanol per 1 ml Trizol, was added to wash RNA pellet by vortexing and centrifugation at 7,500x g for 5 min at 2–8°C. Finally, RNA pellets were dried for 10 min at room temperature (RT). RNA was eluted with 30 μl of RNase-free water, incubated for 10 min at 55°C and kept at -70°C until used.

The organic phase from the previous step was used for isolation of DNA according to the manufacturer’s instructions. The organic phase was mixed with 0.3 ml ethanol per 1 ml Trizol and DNA was mixed and precipitated by incubation for 15–30°C for 2–3 min and centrifuged at 4,000x g for 5 min at 2–8°C. The phenol-ethanol supernatant was removed. Then, 1 ml 0.1 M sodium citrate was added for washing DNA. The DNA pellet was kept in the washing solution for 30 min at 15–30°C and centrifuged at 4,000x g for 5 min at 2–8°C. Next, the DNA pellet was air dried for 15 min, 50 μl of 8 mM NaOH added and stored at -20°C until used.

### HPV DNA detection by polymerase chain reaction (PCR)

The detection of HPV DNA from normal cervical cells, fresh cervical biopsies, and FFPE samples were processed with PCR using GP5+/6+ primers for detection of *Late* gene 1 (*L1*) and PCO4/GH2O primers for detection of the *β-globin* gene (Table 1). The PCR master mix included; 1x PCR buffer, 3.5 mM MgCl_2_, 0.2 mM dNTP, 0.4 mM GP5+ primer, 0.4 mM GP6+ primer [35], Taq DNA polymerase (Thermo scientific, Pittsburgh, PA, USA), DNA template 50–100 ng and distilled water to 25 μl. The PCR condition was as follows; pre-denaturation 5 min at 94°C and 40 cycles of 1 min denaturation at 94°C, 1 min annealing at 42°C, 1 min extension at 72°C and then 4 min final extension at 72°C. The positive cases of HPV DNA were identified by the detection of an *L1* PCR product of approximately 150 bp size. The *β-globin* gene as an internal control was approximately 268 bp sizes [36]. The PCR product was detected using 2.0% agarose gel electrophoresis in 0.5x TAE buffer at 100 V for 27 min.

**Table 1:** Primer sequences.

Gene	Primer sequence	Amplicon (bp)	Ref.
**HPV *L1***	Forward: 5’-TTTGTTACTGTGGTAGATAC TAC-3’	150	35
	Reverse: 5’-GAAAAATAAACTGTAAATCATATTC-3'		
***β-globin***	Forward: 5′-GAAGAGCCAAGGACAGGTAC-3’	268	36
	Reverse: 5′-CAACTTCATCCACGTTCACC-3’		
***PDCD4***	Forward: 5’-GATTAACTGTGCCAACCAGTCCAAAG-3’	150	37
	Reverse: 5’-CATCCACCTCCTCCACATCATACAC-3’		
***IL-6***	Forward: 5’-CTTCGGTCCAGTTGCCTTCT-3’	86	
	Reverse: 5’-TGGAATCTTCTCCTGGGGGT-3’		
α**-*SMA***	Forward: 5’-AGGTAACGAGTCAGAGCTTTGGC-3’	199	25
	Reverse: 5’-CTCTCTGTCCACCTTCCAGCAG-3’		
***GAPDH***	Forward: 5’-TCATCAGCAATGCCTCCTGCA-3’	117	38
	Reverse: 5’-TGGGTGGCAGTGATGGCA-3’		

### Quantitative real-time RT-PCR analysis

TaqMan MicroRNA Reverse transcription reactions and TaqMan MicroRNA quantitative polymerase chain reactions (qPCR) were performed to detect *miR-21* and an endogenous control, RNA *U6* small nuclear (*RNU6B*) expression using the MicroRNA TaqMan Reverse Transcription Kit and the TaqMan MicroRNA Assays (Applied BioSystems, Carlsbad, CA, USA) according to manufacturer’s instructions. The miRNA detection conditions were: 95°C for 10 min and 40 cycles of 95°C for 15 s, 60°C for 1 min. The miRNA expression levels were calculated as the cycle threshold (-delta CT) of *miR-21* and normalized with an endogenous control. The extracted RNA from the HeLa cell line was used as a positive control. Reverse transcription (RT) reactions and quantitative polymerase chain reactions (qPCR) were performed using the Super Script VILO Synthesis Kit and the SsoAdvanced SYBR Green Supermix (Bio-Rad, Hercules, CA, USA). The amount of mRNA targeting gene *PDCD4* [37], signal transducer *IL-6* and fibroblast differentiation marker α*-SMA* were detected (Table 1). The expression of mRNA was normalized to the level of glyceraldehyde 3-phosphate dehydrogenase (*GAPDH*) [38] and calculated as the cycle threshold (2^-delta delta CT^) of the mRNA detection. The conditions were: 95°C for 1 min and 40 cycles of 95°C for 10 s, 60°C for 1 min.

### MicroRNA in situ hybridization

Six μm-thick paraffin sections were mounted on Superfrost glass slides and de-paraffinized. The steps of *in situ* hybridization were as follows: Pre-hybridization step; the slides were treated with 15 μg/ml proteinase-K for 7.5 min at 37°C, then immersed into 3% hydrogen peroxide (H_2_O_2_)/phosphate buffered saline (PBS) for peroxidase blocking for 15 min and dehydrated in new ethanol solutions. Hybridization step; 50–100 μl hybridization mix 50 nM double-DIG LNA *microRNA-21* probe (positive control test: 1 nM LNA *U6* snRNA probe; negative control test 50 nM Scramble probe) (Exqion, Vedbeak, Denmark) was applied onto the slides. The slides were incubated overnight at 56°C (StatSpin ThermoBrite oven, Abbott Molecular Abbott Park, IL, USA), then washed twice with 5x SSC buffer for 5 min at RT; slides were then treated by 5 min stringent wash steps at 56°C in 1x SSC buffer twice followed by 0.2x SSC buffer once. Then, the slides were immersed into 0.2x SSC buffer for 5 min at RT, and incubated with blocking solution (Roche, Mannheim, Germany) containing 2% sheep serum in a humidifying chamber at RT for 15 min. Sheep anti-DIG-horseradish peroxidase (HRP) conjugate (Roche, Mannheim, Germany) was applied diluted at 1:50 for 15 min at RT. Digoxogenin-labeled tyramide [39] was then applied at 1:50 dilution for 30 min at RT, followed by the incubation with sheep anti-DIG-HRP conjugate at 1:100 for 30 min at RT. The hybridization signal was demonstrated using the HRP substrate 3-amino-9-ethylcarbazole (AEC) (Dako, Carpinteria, CA, USA) for 5 min, incubated at RT and counterstained with hematoxylin for 5 min at RT. Finally, the slides were mounted directly with 1–2 drops of aqua mount (Polysciences, Warrington, PA, USA) and air-dried. The image analysis was performed using a Leica microscope (Leica, Herlev, Denmark) fitted with 20x-60x objectives.

### Laser capture microdissection (LCM)

Two or three sections of 3–10 μm-thick FFPE cervical tissues were mounted onto a slide under RNase-free conditions. The procedures were as follows: a deparaffinization step by serial immersion through xylene and graded ethanol washes followed by staining with hematoxylin and eosin; tissue fraction step by selection of 20–100 x 10^3^ μm^2^ (4–6 areas) of tumor stromal or cervical epithelium/cancer cells isolated into separate cups using the PALM Carl Zeiss MicroImaging Laser Capture Microdissection system (Carl Zeiss Microscopy, Jena, Germany) and used for total RNA extraction using a Trizol reagent kit (Ambion Life Technology, Carlsbad, CA, USA) as described above.

### Treatment of fibroblasts

The HPV18-positive HeLa cell line was cultured in DMEM medium (Gibco, life technology, Grand Island, NY, USA) for 48 h. Human primary fibroblasts were sequestered from leftover amniotic fluid cell culture taken by amniocentesis and used for evaluation of prenatal diagnosis of chromosomal abnormalities. The fibroblasts were taken anonymously following laboratory report without any identifying information and without a link to a specific participant or donor. The fibroblasts were subcultured and used after growing more than 95% confluent under microscope. The procedures were approved by the Human Research Ethics Committee of Khon Kaen University (HE571157). The requirement for written informed consent was waived by the committee for reasons including the use of leftover specimens without any identifying information and without a link to a specific participant or donor. These cells were cultured at 37°C in a 5% CO_2_ atmosphere and were treated with culture media (CM) from HeLa cell line. The fibroblast differentiation was evaluated using α*-SMA* as the differentiated marker by qRT-PCR. The expression of *miR-21* and the functional target gene *PDCD4* were measured using qRT-PCR at 24–48 h after treatment with CM and 2 ng/ml recombinant human TGF-β1 (Gibco/Invitrogen, Carlsbad, CA, USA) [25]. In addition, *IL-6* that acts as signal transducer of *miR-21* and during myofibroblast conversion was examined using qRT-PCR. To confirm the expression of *miR-21* in fibroblasts, exosome was isolated from 100 ml HeLa CM and fetal bovine serum (FBS) by ultracentifugation twice at 70,000x g for 30 min as well as form HeLa cell lysate. The exosome pellet was determined for CD63 (exosome marker) and cytochrome C (cellular marker) by western blot using anti-CD63 antibody (clone ab8219, Abcam, Cambridge, England) and anti-cytochrome C antibody (clone ab110325, Abcam, Cambridge, England). The miRNA in the isolated exosome was detected by qRT-PCR.

### Statistical analysis

Data were expressed as mean ± S.D. The results were analyzed with one-way ANOVA or Kruskal-Wallis test for differences among groups of multi-data. The differences between two groups were evaluated by the Student’s *t*-test or Mann-Whitney *U* test. The relative expressions of *miR-21* in cervical tissues were calculated by the equation:-delta CT = —(CT_*miR-21*_-CT_*U6*_). The relative expression of RNA in the *in vitro* study was calculated as the 2^-delta delta CT^, after normalizing with the housekeeping gene (GAPDH) and relative to the untreated control. Statistical significance was considered a *P*-value <0.05 using SPSS software.

## Results

### HPV infection and *miR-21* expression in cervical tissues

**Fig 1:**
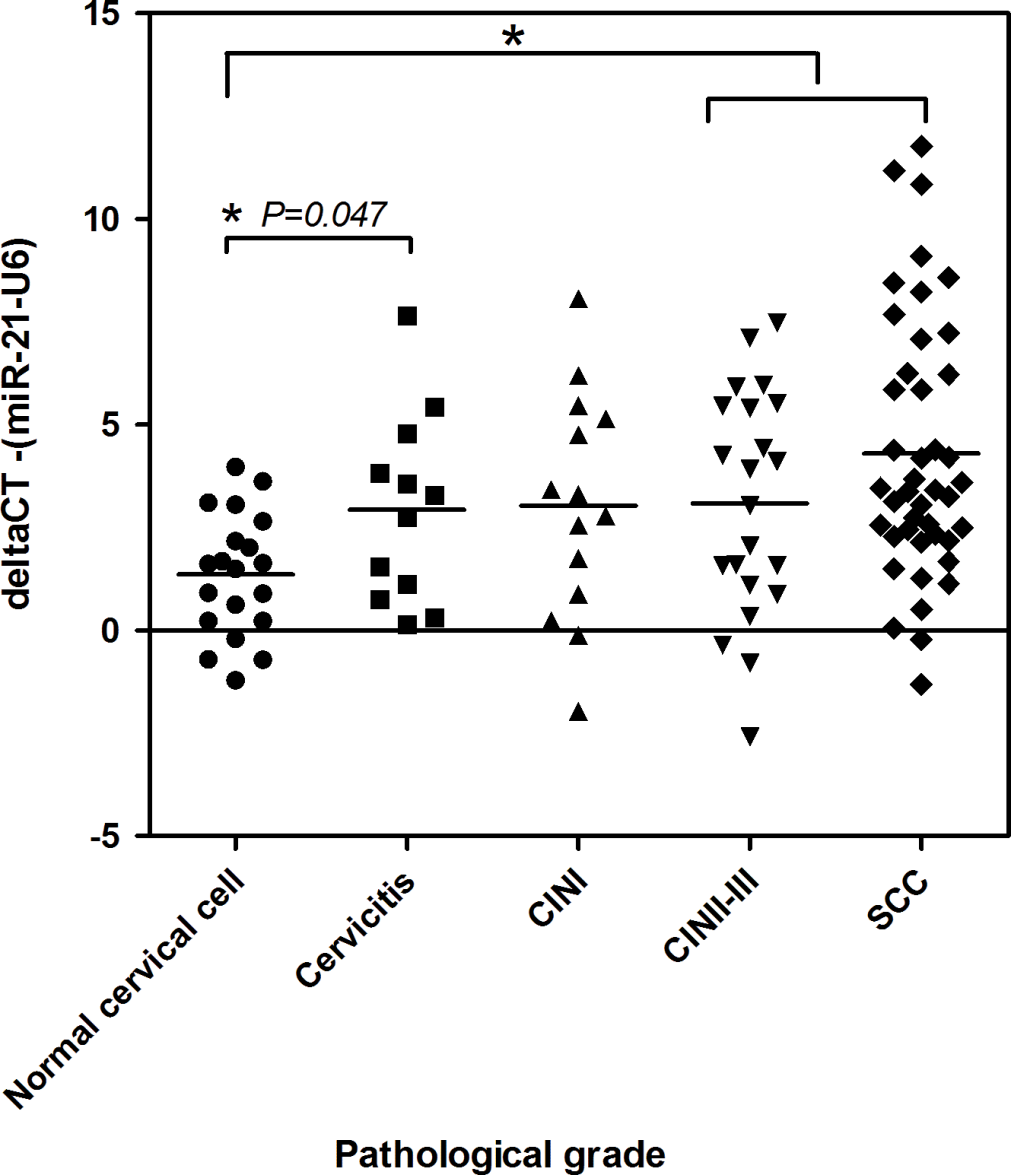
The *miR-21* expression levels of fresh cervical biopsy samples in relation to histological grading. *miR-21* expression levels were measured by qRT-PCR. Significant differences between pathological grades and *miR-21* expression (-delta CT) were found at *P* = 0.005. The expression levels of *miR-21* were significantly lower in normal cervical cells than in cervicitis, CIN II-III and SCC (*Student’s T-Test, *P* = 0.025, *P* = 0.014 and *P* = 0.000).

A total of 111 fresh specimens were available for *miR-21* expression analysis. These specimens included 20 normal cervical cell, 12 cervicitis, 14 CIN I, 22 CIN II-III, and 43 SCC samples. The expression levels of *miR-21* calculated as ∆CT were lower in normal cervical cells (Mean = 1.35±S.D. = 1.49) than in CIN I (3.03±2.75), CIN II-III (3.09±2.73) and SCC (4.30±3.15) (*P* = 0.052, *P* = 0.014 and *P* = 0.000, respectively) (Fig 1).

**Fig 2:**
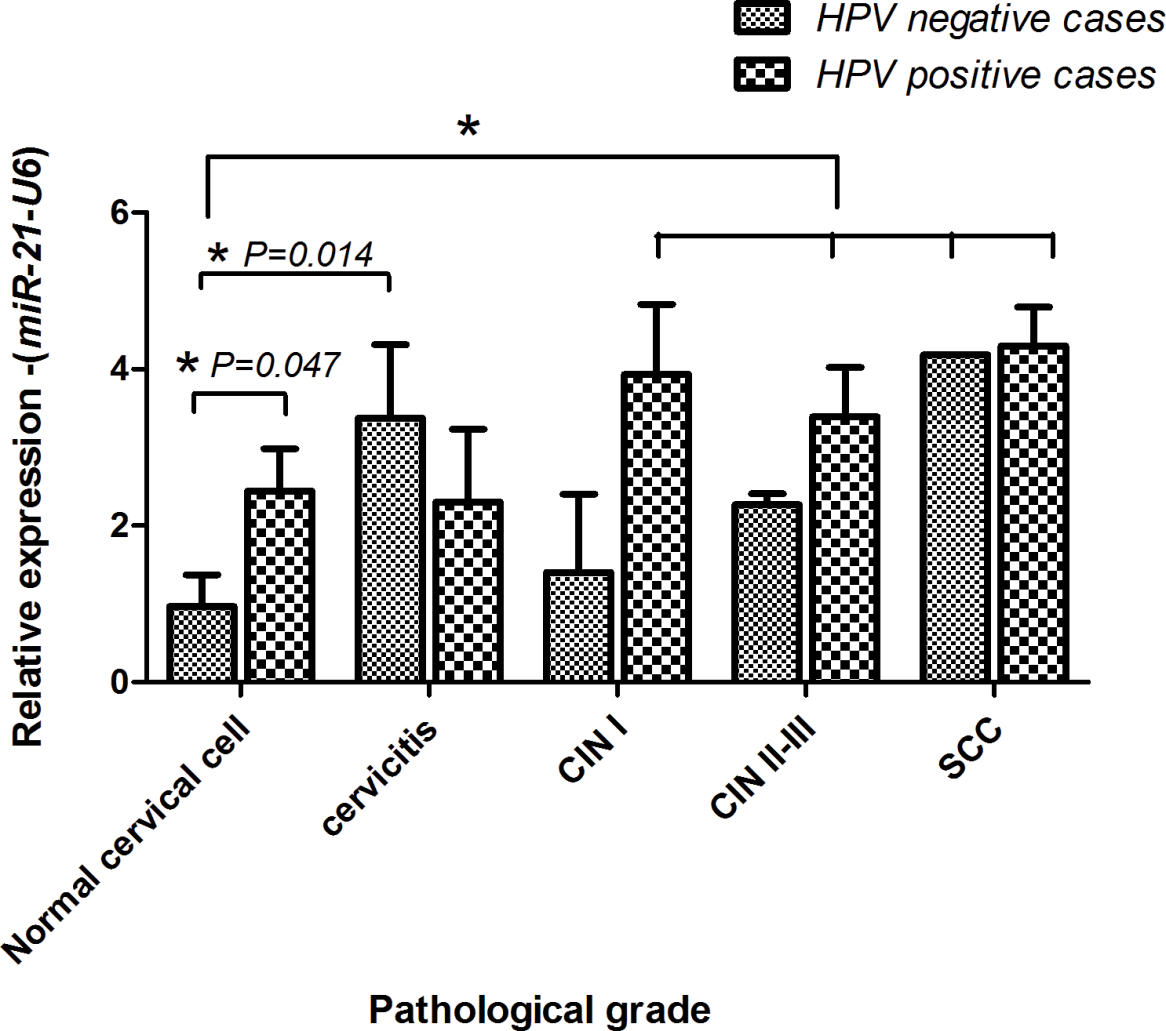
The characteristics of *miR-21* expression in cervicitis, CIN I, CIN II-III and SCC. The level of transcriptional change was examined by qRT-PCR. HPV positive rates and *miR-21* expression levels in relation to cytological stages. (*: significant difference from control at *P*<0.05)

HPV DNA was detected in 40% (8/20 cases) of normal cervical cells, 50% (5/12 cases) of cervicitis, 64.2% (9/14 cases) of CIN I, 72.7% (16/22 cases) of CIN II-III and 79% (34/43 cases) of SCC. The expression of *miR-21* in normal cervical cells was significantly higher in HPV positive cases (1.93±1.52) than HPV negative cases (0.97±1.40, *P* = 0.047). In addition, the expression of *miR-21* in HPV negative normal cytology (0.97±1.40) was significantly lower than HPV positive samples in worsening histopathological grades (Kruskal-Wallis Test, *P* = 0.003); CIN I (3.93±2.68, *P* = 0.011); CIN II-III (3.39±2.50, *P* = 0.003) and SCC (4.37±3.24, *P* = 0.000) (Fig 2). HPV positive cases of CINs and SCC showed higher levels of *miR-21* than HPV negative cases, but not significantly (Fig 2). A significant down-regulation of *PDCD4*, which is the targeting gene of *miR-21*, was found and associated with the up-regulation of *miR-21* status (*P* = 0.000).

### *miR-21* expression in cervicitis

The expression of *miR-21* was up-regulated in cervicitis (2.93±2.29) and significantly higher than normal cervical cells (1.35±1.49, *P* = 0.025) (Fig 1). Interestingly, the level of *miR-21* was higher in HPV negative cervicitis (3.37±2.49) than HPV negative normal cervical cells (0.97±1.39, *P* = 0.014) whereas no differences were found between the HPV positive cases of normal cervical cells and cervicitis cases (Fig 2).

### ISH detection for the *miR-21* in the cervical cancer tissues

**Fig 3:**
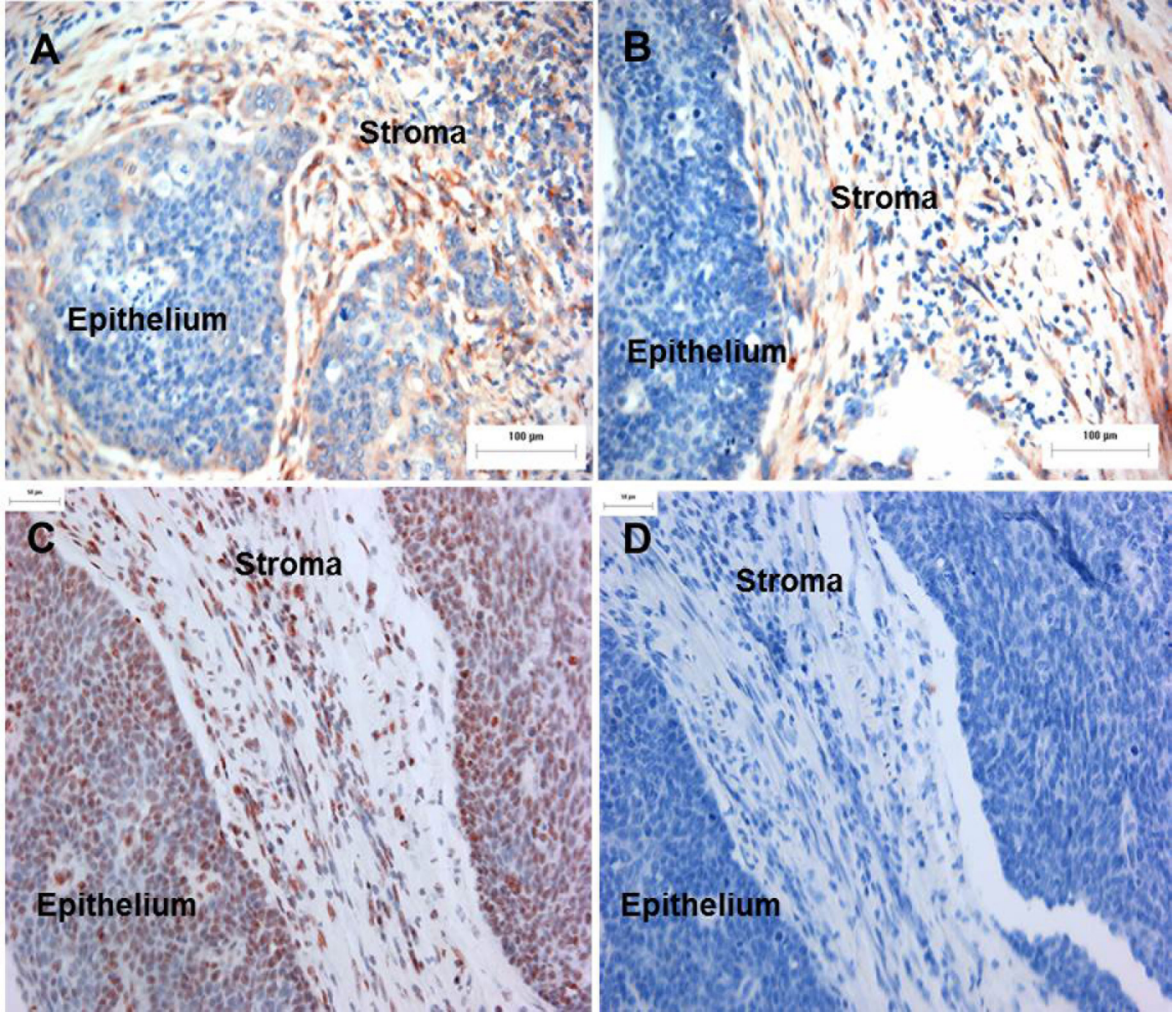
The localization of *miR-21* in invasive cervical cancer tissues. A and B: tissue sections from cervical cancer samples were incubated with DIG-labeled locked nuclei acid (LNA) probe to *miR-21*. Strong signals were observed in the stroma. The signal was also seen at the rim of cancer cells adjacent to the stroma (Bar: 100 μm). C: *RNU6B* staining in cervical cancer and stromal nuclei (Bar: 50 μm). D: cervical cancer negative control staining using a ‘scramble’ LNA probe (Bar: 50 μm).

To understand the up-regulation of *miR-21* expression in cervical cancer tissues, 5 cases of FFPE SCC samples were sectioned and *miR-21* expression was detected by ISH. The results showed that the *miR-21* expression was predominantly detected in tumor associated stroma of SCC. In the cancer tissues, positive signals were seen mostly at the invasive front near the stroma (Fig 3A and 3B). Fig 3C shows staining of tumor and stromal nuclei for *RNU6*. Fig 3D shows negative staining with a scramble probe.

### Localization of *miR-21* expression in FFPE cervical tissues

To confirm ISH observations, the expression of *miR-21* in the cancer cells and the stroma were separately measured in all 70 FFPE cervical tissue samples including cervicitis tissues using qRT-PCR. The results showed that the *miR-21* was up-regulated in the tumor-associated stroma of CIN II-III and SCC, corresponding with the histopathological grades. The results showed that the expression level of *miR-21* in tumor (-3.19±5.75) was less than in tumor-stroma (3.00±1.4, *P* = 0.045) (Table 2).

**Table 2:** Comparative expressions of *miR-21* in the epithelial and stromal regions of 70 FFPE cervical tissues.

*miR-21* (-∆CT) N = 70	Cervicitis	CIN I	CIN II-III	SCC
	N = 26	N = 11	N = 21	N = 12
Epithelium	-0.40±4.02	-0.39±6.64	-0.35±3.05	-3.19±5.75
Stroma	1.68±1.83	-1.60±4.10	1.75±1.16	3.00±1.44
*P-value*	0.236	0.892	0.244	0.045

Cervical intraepithelial neoplasia I (CIN I), Cervical intraepithelial neoplasia II and III (CIN II-III), cervical squamous cell carcinoma (SCC). A significant difference was found between epithelium and stroma of SCC (*P*<0.05).

### *miR-21* expression in the activated fibroblasts

**Fig 4:**
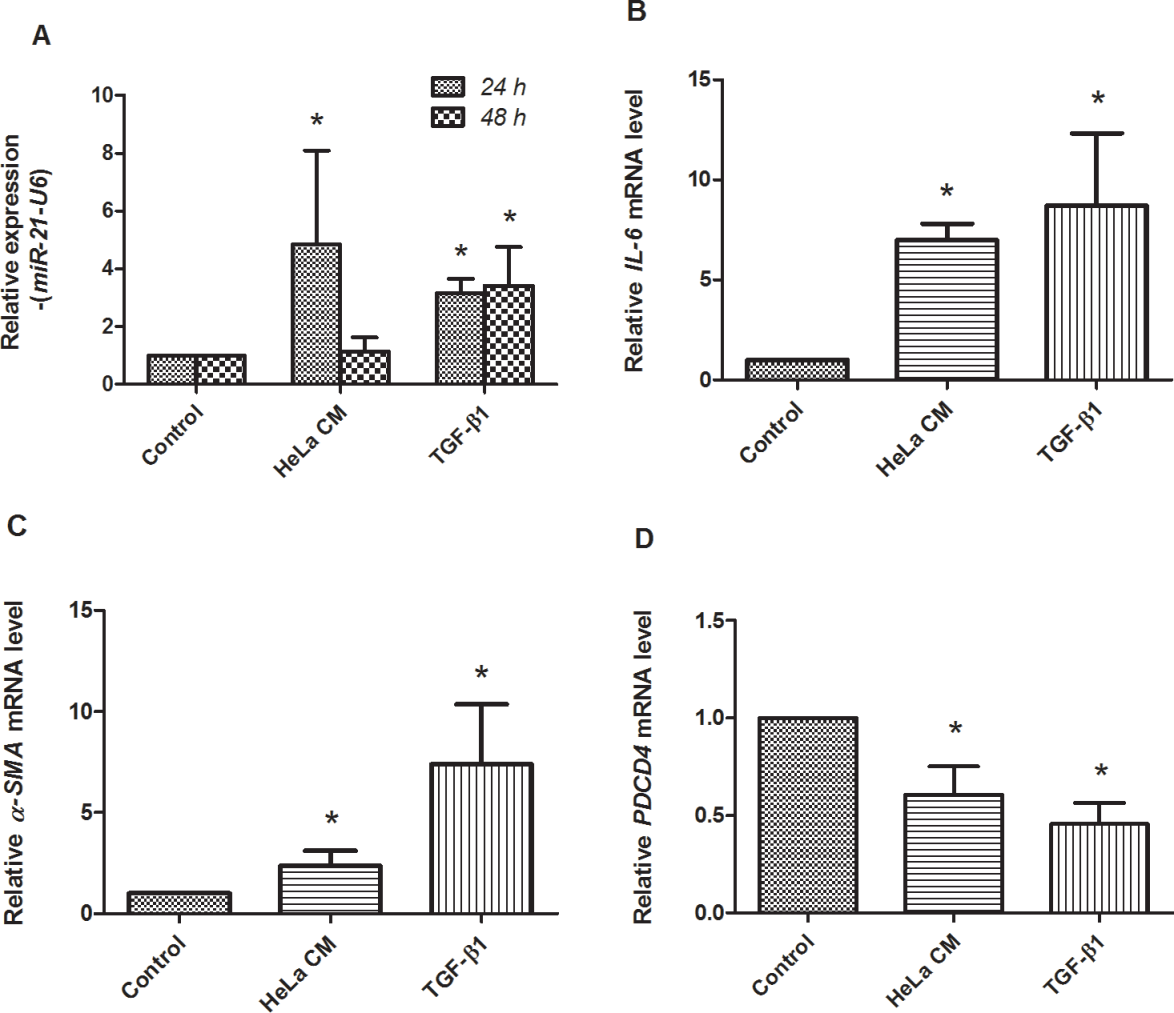
The characteristics of stromal fibroblasts after treatment with culture media from HeLa cells (HeLa CM) and TGF-β1. The level of transcriptional change was examined by real-time RT-PCR. A: the expression of *miR-21* was significantly up-regulated at 24 and 48 h after HeLa CM or TGF-β1 treatment. B and C: the expression of *IL-6* and alpha-*SMA* was significantly up-regulated at 48 h after HeLa CM or TGF-β1 treatment. D: *PDCD4* was significantly down-regulated at 48 h after HeLa CM or TGF-β1 treatment. (*: significant differences from control at *P*<0.05)

**Fig 5:**
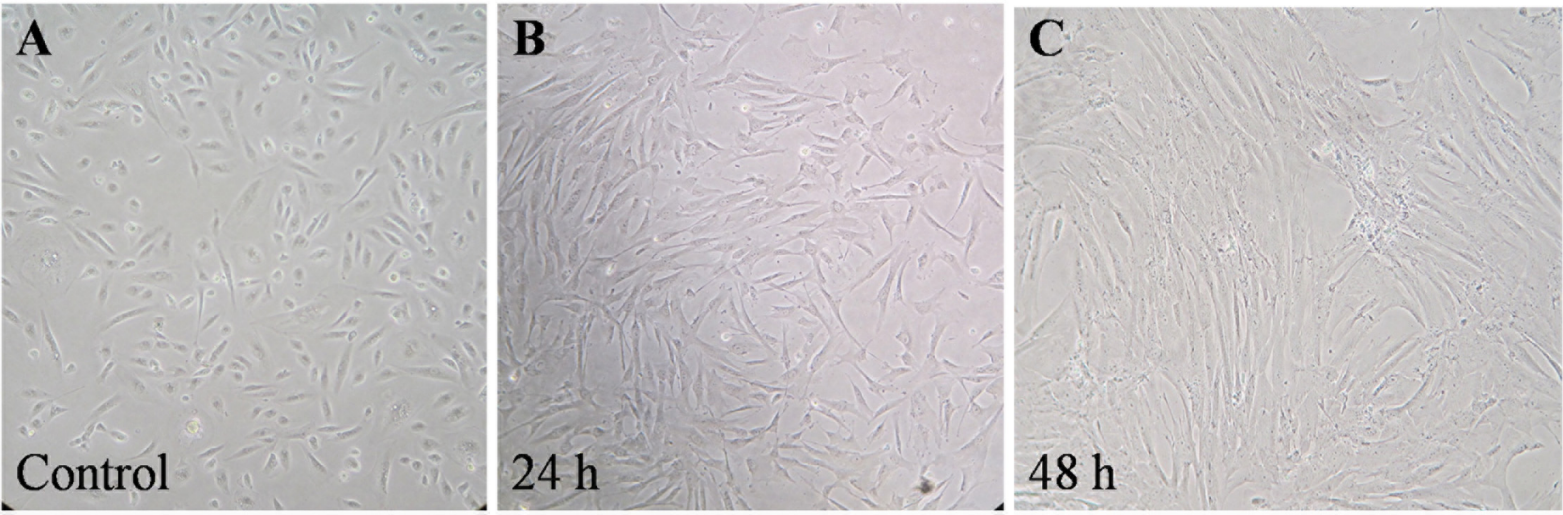
The morphology of fibroblasts treated with culture media from HeLa cells. HeLa cells were cultured in exosome-depleted media. The treated cells were elongated.

Culture media from HPV positive HeLa cells (HeLa CM) is involved in the activation of *miR-21* over-expression in fibroblasts [25]. To investigate the role of HPV infection on the up-regulation of *miR-21* in the stromal cells, fibroblasts were treated with HeLa CM and TGF-β1 was used as the positive control. The levels of *miR-21* expression were measured by qRT-PCR at 24–48 h. The results are shown in Fig 4. After 24-h treatment with HeLa CM and TGF-β1, the *miR-21* levels were significantly 4.85- and 3.15-fold higher than untreated controls (*P* = 0.029 and 0.029) (Fig 4A). A decrease in *miR-21* expression was found in fibroblasts at 48 h after HeLa CM treatment. The *IL-6* expression as a signal transducer of *miR-21* [26] was also determined in the treated fibroblasts. Fig 4B shows the levels of *IL-6* mRNA expression that are significantly up-regulated by 6.99 and 8.71 fold (*P* = 0.002 and 0.024) in fibroblasts treated with HeLa CM and TGF-β1, for 48 h, compared to the untreated controls. These results demonstrated that the up-regulation of *miR-21* was associated with *IL-6* expression. In addition, α*-SMA*, a myofibroblast differentiation marker was also up-regulated by 2.35 and 7.40 folds (*P* = 0.002 and *P* = 0.010) at 48 h in fibroblasts treated with HeLa CM and TGF-β1 (Fig 4B and 4C). The myofibroblasts, which are known to express *miR-21* and drive tumorigenesis [25], are induced by numerous cytokines including TGF-β. To confirm the expression of *miR-21* in the treated fibroblasts, the levels of *PDCD4* expression were investigated. The results showed that *PDCD4* expression was significantly down-regulated as shown in Fig 4D. Fig 5 shows morphology of the treated fibroblasts at 24 and 48 h after treatment. These data demonstrated the up-regulation of *miR-21* in myofibroblasts that may be associated with HPV infected cervical cells. This result supported that *miR-21* was detected in HeLa cells but not in exosome extracted from HeLa CM and fetal bovine serum (FBS). In addition, we also detected *miR-21* expression in fibroblasts treated with culture media from HeLa cultured in exosome-depleted FBS media (10% exosome-free FBS in DMEM) and the result was quite similar to the experiment using culture media from HeLa cultured in conventional cell culture media (data not shown). This result confirmed that *miR-21* was not from HeLa CM but was expressed in the HeLa CM-treated fibroblasts.

## Discussion

*miR-21* is a target of considerable interest because of its up-regulation demonstrated by qRT-PCR in a variety of human cancers including cervical tumors [7–9, 14, 16, 19, 25]. In this study, *miR-21* expression in normal, cervicitis, CIN and SCC was investigated by combining qRT-PCR, microdissection and *in situ* hybridization. HPV infections were also investigated. The relationship of *miR-21* to *α-SMA*, *IL-6 and PDCD4* expression was examined in a fibroblast cell differentiation model.

In this study, prevalence of HPV in normal was quite high (40%) that may be affected from a small sample size, sampling method, specimen collection and HPV detection technique. The cervical cell samples were taken from women who underwent cervical cancer screening and collected in ThinPrep test vials. HPV detection was performed by PCR using Q5 High-Fidelity DNA Polymerase which is ideal for difficult amplicons.

The results showed the up-regulation of *miR-21* corresponded with histological grades when compared with HPV negative normal cytology; this finding is similar to a study by Deftereos et al. who found that *miR-21* expression had a significant correlation with histological changes ranging from normal to a worsening histological diagnosis of cervical cancer [19]. In these experiments, the expression of *miR-21* was shown to be significantly associated with HPV infection. In addition, the studies found that the expression of *miR-21* was significantly up-regulated in the transition from normal to cervicitis corresponding with down-regulation of *PDCD4*. This may represent an important event in the role of *miR-21* in carcinogenesis and an inflammatory response such as: First, by down-regulation of molecule *PDCD4* [40] resulting in suppression of the inflammation process via transcriptional factor NF-kB and activated anti-inflammatory cytokine, interleukin 10 (IL-10) [41, 42] leading to immune evasion and cervical cancer progression. Second, by down regulated *PTEN* and activated Akt signaling pathways that increased NF-kB activity leading to inflammation as described by Lliopoulos et al., 2010 who studied colon cancer and suggested that epigenetic switch of untransformed cells to transformed cells links the inflammation to cancer [20]. The present studies demonstrate that the induction of *miR-21* might be involved in HPV infection, and the inflammation process of cervicitis.

The microdissection/qRT-PCR and ISH data show for the first time that *miR-21* expression in cervical tumors may occur primarily in the tumor-associated stromal microenvironment rather than directly in tumor cells themselves (Fig 3). This finding accords with recent colorectal cancer studies (that included ISH), which have found *miR-21* up-regulation in the stromal compartment rather than in tumor cells, and associated with poor disease free survival [14]. The tumor-stromal cells of cervical cancer that expressed *miR-21* generally showed fibroblast-like morphology. Most cancer associated fibroblasts constitute a heterogeneous cell population and differentiate to cells called myofibroblasts which promote tumor progression via production of growth promoting factors [23]. Fibroblasts may act on tumor cells through expression of growth factors, such as TGF-β1, and thereby contribute to the survival and proliferation of tumor cells. TGF-β1 activated the Erk-MAP kinase pathway leading to activation of AP-1 expression that acts as a putative activator of *miR-21* promoter in stromal fibroblasts and in cancer cells of an advanced stage [25, 40, 43]. By qRT-PCR, this present study demonstrated that expression of *miR-21* was up-regulated in fibroblasts treated with HeLa CM or TGF-β1 (Fig 4). However, a decrease of *miR-21* was shown in fibroblasts treated with HeLa CM for 48 h. This result is similar to the result of the previous study [25]. It might be that there was a limited amount of TGF- β1 in HeLa CM compared with culture media supplemented with TGF-β1. Treatment with HeLa CM and TGF-β1 also showed significant up-regulation of α*-SMA*. This result suggested differentiation of fibroblasts to myofibroblasts (Fig 4). *miR-21* might have a potential role in gene regulation during promotion of fibroblast differentiation. These findings suggest that up-regulation of *miR-21* may contribute to the development of carcinogenesis.

The results of the current study also showed the over-expression of *IL-6* mRNA in fibroblasts after treatment with HeLa CM and TGF-β1 (Fig 4). *IL-6* mRNA was up-regulated during fibroblast differentiation observed by α*-SMA* detection. This suggested an involvement of the *IL-6* signaling pathway in up-regulation of *miR-21* in the stroma. Iglesias et al. showed that cervical fibroblasts constitutively secreted *IL-6* in significantly greater amounts than normal cervical epithelial cells [44]. Kinoshita et al. reported that *IL-6* mediates epithelial-stromal interactions and promotes gastric tumorigenesis [26]. Tartour et al. also showed that IL-6 protein detected by immunohistochemistry (IHC) was only found in the cervical stromal cells [34]. The results in this study confirm that up-regulation of *miR-21* expression is mostly found in cervical stromal lesion. Wei et al. showed that IL-6 protein was expressed in most basal and parabasal cells of normal epithelium and invasive squamous cell carcinoma of the cervix by IHC [27]. The present study found the up-regulation of *miR-21* in both stroma and the rim of cervical cancer cells near the stroma. These findings support the idea that fibroblasts are a significant source of *miR-21* expression and *IL-6* production, and HPV positive cervical cancer cells play a crucial role in the induction of *IL-6* mRNA expression and up-regulation of *miR-21* in stromal fibroblasts.

In these studies it was found that the expression of *miR-21* was associated with HPV-infection in cervical lesions and cervicitis. In addition, the studies herein showed the expression of *miR-21* in tumor-stromal of SCC and an association with fibroblast differentiation and warrants further studies.
